# LncRNA NONRATT021972 Was Associated with Neuropathic Pain Scoring in Patients with Type 2 Diabetes

**DOI:** 10.1155/2017/2941297

**Published:** 2017-08-08

**Authors:** Wei Yu, Guo-qing Zhao, Rang-juan Cao, Zhi-hua Zhu, Kai Li

**Affiliations:** ^1^Hand-Surgery Department, China-Japan Union Hospital of Jilin University, Changchun, China; ^2^Anesthesia Department, China-Japan Union Hospital of Jilin University, Changchun, China

## Abstract

**Background:**

Long noncoding RNAs were involved in the processes of diabetes. Our study was aimed to explore clinical potential of LncRNA NONRATT021972 in diabetic neuropathic pain and investigate detailed mechanisms.

**Methods:**

154 patients with type 2 diabetes were enrolled as experimental group paired with control. Patients without diabetes but neuropathy were enrolled to explore exclusive role of LncRNA NONRATT021972 in neuropathy. Real-time PCR and ELISA were performed to examine expression of LncRNA and TNF-*α* in flood. Neuropathic pain scores were calculated with data from NPQ. Streptozotocin was used for SD adult male rats to establish diabetes for NONRATT021972 siRNA or saline treatment. Neuropathic pain behaviors and expression of TNF-*α* were assessed.

**Result:**

Patients with type 2 diabetes had a significantly higher concentration of LncRNA NONRATT021972 in blood and more severe symptoms of neuropathic pain. LncRNA NONRATT021972 was positively associated with neuropathic pain scores of type 2 diabetes. TNF-*α* level increased in patients with type 2 diabetes. Animal experiment showed that LncRNA NONRATT021972 siRNA attenuated inflammation via decreasing TNF-*α* and alleviated neuropathic pain.

**Conclusion:**

LncRNA NONRATT021972 increased in type 2 diabetes and was positively associated with neuropathic pain scoring in type 2 diabetes. LncRNA NONRATT021972 exacerbated neuropathic pain via TNF-*α* related pathways.

## 1. Introduction

Long noncoding RNA (LncRNA) belongs to a family of small RNAs and is characterized with transcripts that are >200 nucleotides in molecular length [1]. LncRNAs can be transcribed, ranging from 195 bp up to a couple of kilobases in length according to the sequence of LncRNA nearby protein-coding genes [2]. Previous studies have indicated that LncRNA is involved in the occurrence and progression of diabetes and produces a complex regulatory network through interactions with transcription factors in type 2 diabetes [3, 4]. On the other hand, LncRNAs are also involved in the pathological progression of the nervous system and have multiple effects on neuropathic pain [5].

Diabetes mellitus has become a global health issue in recent years, with a global incidence of 14.6% [6]. In a clinical scenario, neuropathic pain is one of the most common chronic complications in patients with type 2 diabetes, and character with typical symptoms of pathological pain, including hyperalgesia, spontaneous pain, and specific allodynia [7–9]. Clinical studies showed that the intractable pain of diabetes mellitus has become a substantial problem and exacerbated prognosis [10, 11]. Epidemiologic studies showed that more diabetic patients suffered from neuropathic pain [12]. Studies have also suggested that neuropathic pain is correlated with the level of inflammation in patients with type 2 diabetes, while there is no promising therapy for the alleviation of such neuropathic pain [13, 14]. Thus, it is urgent to develop a novel therapy for neuropathic pain of type 2 diabetes due to its adverse effects and the enormous number of diabetes patients.

NONRATT021972 is an LncRNA which was proved with a diabetes-promoting effect, and its sequence was well determined (http://www.noncode.org/show_rna.php?id=NONRATT021972). Animal experiments have shown that expression of NONRATT021972 increased in diabetic mice, and participated in the transmission of nociceptive signaling, especially in neuropathic pain [15]. What is more, there are data suggesting that NONRATT021972 silence was related to decreased inflammation; however, although researchers found that LncNONRATT021972 could regulate P2X7 and P2X3 receptors in dorsal root ganglia, no detailed mechanisms were explored in the field of inflammation [16]. In the clinical application, it is unclear whether LncNONRATT021972 could be a predictor for type 2 diabetes, and there is also no report about how LncNONRATT021972 influenced neuropathic pain of type 2 diabetes in the clinical scenario. Intriguing, bioinformatics data indicated that TNF-*α* might be influenced by NONRATT021972, and TNF-*α* was an important factor in the progress of inflammation.

Accordingly, we hypothesized that LncNONRATT021972 could be a potential biomarker for neuropathic pain of type 2 diabetes in the clinical scenario, and TNF-*α* was a potential downstream factor under regulation of LncNONRATT021972.

## 2. Method and Materials

### 2.1. Clinical Samples

154 patients with type 2 diabetes were enrolled as the experimental group from a local hospital. All patients signed informed consent and consent with following examinations. The inclusion criteria were as follows: male, aging 55 to 65 years, with type 2 diabetes, no history of hypertension, and no history of chronic kidney disease. The same number of healthy subjects was enrolled from the same hospital as the control group, which are matched to the experimental group with similar baseline states.

To further investigate whether LncNONRATT021972 is exclusively a biomarker of pain, we enrolled patients without diabetes but have neuropathy. The inclusion criteria were as follows: patients with confirmed neuropathic pain, especially patients with polyneuropathy (i.e., distal symmetric sensory polyneuropathy patients, small fiber neuropathy patients, or localized neuropathy patients) and patients without diabetes. The diagnosis standard of neuropathy is in accordance with the statement of the American Diabetes Association. Distal symmetric sensory polyneuropathy group (DPN group) had 76 samples, while small fiber neuropathy group (SFN group) had 46 samples, and localized neuropathy group (LN group) had 65 samples.

Blood samples harvested from both groups were collected and allowed to coagulate for 30 min at room temperature. All samples were centrifuged for 10 min (1300 ×g). The collected serum was centrifuged for another 10 min (3000 ×g) to remove any remaining cellular components. Supernatants were transferred into a 500 *μ*L EP tube and stored immediately at −80°C.

### 2.2. Serum Isolation of NONRATT021972 and RT-PCR

Total RNA was isolated from the serum using the RNApure Circulating Reagent (CWBIO, Beijing, catalog number CW2281) with routine protocols. Briefly, serum sample (300 *μ*L) was added three times to volumes of RNApure Circulating Reagent and mixed thoroughly via vortex. Samples were stored to maintain reaction at room temperature for five minutes. Further, 1/5 volume of chloroform was added, mixed vigorously for 30 s, and incubated for 5 min at room temperature. The mixture was centrifuged for 20 min (12,000 ×g, 4°C). The supernatant was transferred to a new sterile EP tube, and the same volume of isopropanol was added and mixed thoroughly for 30 min at room temperature. The mixture was centrifuged for 20 min (12,000 ×g, 4°C). The precipitate was transferred and rinsed twice with 1 mL of 75% ethanol (dilute with DD H_2_O). Isolated RNA was eluted by RNase-free water (20 *μ*L) with routine protocols.

Total RNA (1000 ng) was prepared as standard template for reverse transcription with the RevertAid First Strand cDNA Synthesis Kit (Thermo, USA). PCR amplification of NONRATT021972 and GAPDH (control) was performed according to the reported method.

The primers were as follows: NONRATT021972, sense 5-TGTTCGTGCA AACTGTCAGCT-3 and antisense 5-GGGATGGTTCAAAAGCTTCA-3; and GAPDH, sense 5-CAGGGCTGCTTTTAACTCT GGT-3 and antisense 5-GATTTTGGAGGGATCTCGCT-3.

The estimated length of the PCR product was 250 bp and 199 bp for NONRATT021972 and GAPDH, respectively. The thermal cycling protocols were as follows: 95°C for 30 s; 40 cycles of amplification at 95°C for 5 s; and 60°C for 30 s. The results of PCR were analyzed by the software with PCR instrument (ABI7500).

### 2.3. Measurement of TNF-*α*

The TNF-*α* levels of blood samples were quantified with enzyme-linked immunosorbent assay (ELISA), and antibodies used in our experiment were commercially available. The protocol was provided by the ELISA kit supplier (Senxiong Company, Shanghai, China). The reactions were performed and assessed using a standard ELISA reader (Rayto, RT-6000, USA) at 450 nm. The concentrations of TNF-*α* were determined with routine protocol.

### 2.4. Scoring with the Neuropathic Pain Questionnaire

All subjects in two groups received and completed the neuropathic pain questionnaire (NPQ). Data collect from questionnaire were assessed by three independent expertized individuals for neuropathic pain scores. Cronbach's alpha coefficient and Guttman's split-half coefficient were examined to determine the efficacy of NPQ. NPQ scoring was performed according to guideline recommendation.

### 2.5. NONRATT021972 Small Interference RNA Treatment

The small interference RNA (siRNA) of NONRATT021972 was purchased from Invitrogen (Carlsbad, CA). siRNA targeted to NONRATT021972 was used in our experiment. The siRNA was diluted with 80 *μ*L of RNA free water and added with 80 *μ*L of 10% glucose as mixture A. Mixture B was produced by mixing 80 *μ*L of transfection reagent and 80 *μ*L 10% glucose. Mixture B and mixture A were mixed for 15 min reaction. The siRNA target sequence was 5-GAATGTTGGTC ATATCAAA-3 (Invitrogen).

### 2.6. Animal Models of Diabetes and NONRATT021972 siRNA Treatment

40 SD adult male rats were prepared for diabetes model, and intraperitoneal injection of STZ (30 mg/kg) was performed to establish animal models of diabetes with reported protocols. Fasting blood glucose > 7.8 mmol/L and nonfasting blood glucose > 11.1 mmol/L were considered to be a successful diabetes model. 20 diabetic rats were randomly injected with NONRATT021972 siRNA (320 *μ*L) per week.

### 2.7. Behavioral Studies of Diabetic Rats

The thermal withdrawal latency was determined with the Thermal Paw Stimulation System (BME-410C, Tianjin). Rats were placed in a transparent box (50 cm × 35 cm × 50 cm) with glass plate under which a thermal light source was located. After a 30 min adaptive phase, the paw of rats was exposed to a beam of radiant heat applied through the glass floor. Activation of the thermal light source simultaneously activated a timer, and both were immediately turned off by paw withdrawal or at the 25 s cutoff time.

Mechanical withdrawal threshold was assessed by observing withdrawal responses to mechanical stimulation using Von Frey filaments (Stoelting, Wood Dale, IL, USA) with reported protocols.

Both examinations were performed at 4 time points after establishment of diabetic model (1 week, 2 weeks, 4 weeks, and 8 weeks).

### 2.8. Statistical Analysis

SPSS 16.0 software was used for data processing. Measurement data are normal distribution to mean ± SD. *t*-test was performed for data with normal distribution and equal variance. Logistic Linear regression was performed to determine association among variates.

## 3. Result

### 3.1. LncRNA NONRATT021972 Increased in Type 2 Diabetes

**Figure 1:**
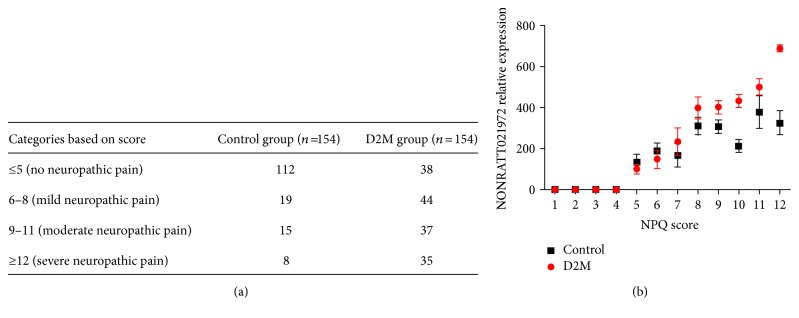
(a) Numbers of patients with different NPQ score in two groups. (b) Association between NPQ score and NONRATT021972 relative expression.

Compared with the control group, serum concentration of LncRNA NONRATT021972 indeed increased in patients with type 2 diabetes (*P* < 0.05), suggesting that increase of LncRNA NONRATT021972 was associated with increased blood glucose or occurrence of type 2 diabetes and LncRNA NONRATT021972 might be a novel biomarker of type 2 diabetes (Table 1). Moreover, NPQ showed that incidence of neuropathic pain significantly increased in the diabetes group (Figure 1) with promising credibility (Table 2), verified by Cronbach's alpha coefficient and Guttman split-half coefficient.

### 3.2. LncRNA NONRATT021972 Was Associated with Neuropathic Pain in Type 2 Diabetes

Further intragroup analysis showed that serum levels of LncRNA NONRATT021972 varied relatively greatly among patients with type 2 diabetes (Table 1), and a similar trend was observed in neuropathic pain scoring, verified by NPQ. Logistic linear regression showed that neuropathic pain scoring was positively related to the level of LncRNA NONRATT021972, suggesting that the pain-aggravation effect of LncRNA NONRATT021972 was concentration-dependent (Figure 1). Moreover, symptom types of neuropathic pain increased in patients with a high level of LncRNA NONRATT021972.

### 3.3. Serum Level of TNF-*α* Increased in Type 2 Diabetes

**Figure 2:**
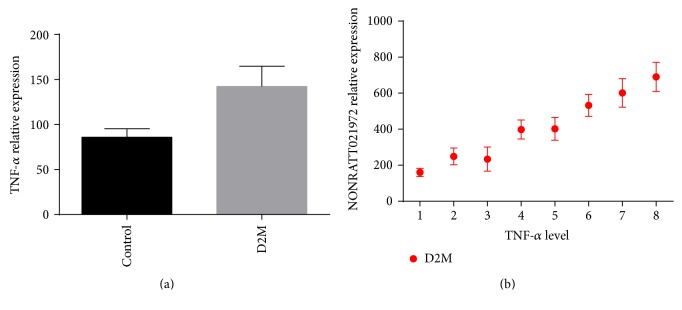
(a) TNF-*α* expression in two groups. (b) Correlation of TNF-*α* expression and NONRATT021972 relative expression.

ELISA showed that, compared with the control group, patients with type 2 diabetes had a remarkably higher level of TNF-*α* (*P* < 0.05, Figure 2(a)), and such increase was associated with levels of LncRNA NONRATT021972 (*P* < 0.05, Figure 2(b)).

### 3.4. LncRNA NONRATT021972 siRNA Decreased TNF-*α* in Diabetic Rats

**Figure 3:**
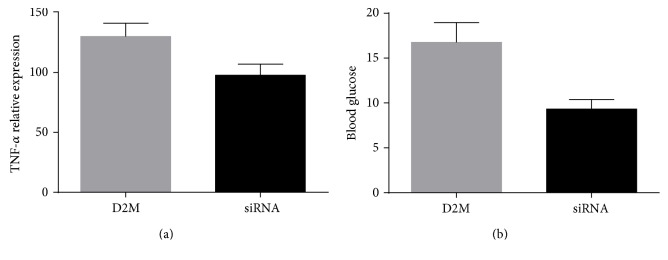
(a) TNF-*α* expression in D2M group and SiRNA group. (b) Blood glucose in D2M group and SiRNA group. SiRNA group, treated by NONRATT021972 siRNA.

STZ-induced diabetes models were successfully established in SD adult male rats. Further, LncRNA NONRATT021972 siRNA decreased blood glucose level and decreased TNF-*α*, verified by ELISA (*P* < 0.05, Figure 3), suggesting that inhibition of LncRNA NONRATT021972 attenuated inflammation of STZ-induced diabetes.

### 3.5. LncRNA NONRATT021972 siRNA Alleviated Neuropathic Pain in Diabetic Rats

**Figure 4:**
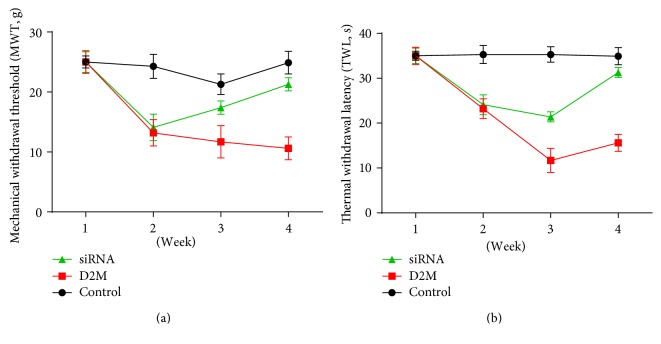
(a) Temporal trend of mechanical withdrawal threshold in three groups. (b) Temporal trend of thermal withdrawal latency in three groups.

Compared with normal rats, STZ-induced diabetic rats had more severe symptoms of neuropathic pain, verified by increased duration of mechanical withdrawal threshold and the thermal withdrawal latency (Figure 4). LncRNA NONRATT021972 siRNA significantly decreased the duration of mechanical withdrawal threshold and the thermal withdrawal latency after 4 weeks treatment (Figure 4), suggesting that LncRNA NONRATT021972 siRNA alleviated neuropathic pain in diabetic rats.

### 3.6. LncRNA NONRATT021972 Was Not Associated with Neuropathic Pain in Patients without Type 2 Diabetes

Further analysis showed that, compared with controls, the level of LncRNA NONRATT021972 did not significantly change in DPN group, and a similar phenomenon was observed in SLN versus control and LN versus control (Table 3).

## 4. Discussion

Although LncRNAs accounts for a quite high proportion of RNA profile in mammal, current studies only revealed a small number of LncRNAs which have been functionally proved, and functions of most LncRNAs are unclear [17]. Recent evidence from animal experiments indicated that the abnormal expression of LncRNAs participates in the occurrence and progression of type 2 diabetes, in which LncRNA ONRATT021972 was a biomarker with promising clinical potential [18, 19].

Our studies have firstly shown that increased serum level of LncRNA NONRATT021972 was positively associated with grade neuropathic pain in patients with type 2 diabetes, suggesting that LncRNA NONRATT021972 was a biomarker or predictive factor for neuropathic pain of type 2 diabetes. Further clinical trials explored that there was no association between neuropathy and LncRNA NONRATT021972 in patients without diabetes, suggesting that LncRNA NONRATT021972 was a biomarker for type 2 diabetes and not exclusively a marker for neuropathy. What is more, increased LncRNA NONRATT021972 could aggravate neuropathic pain via activating TNF-*α*-related pathways in patients with type 2 diabetes. Further animal experiments showed that inhibition of LncRNA NONRATT021972 indeed alleviate neuropathic pain of type 2 diabetes via decreasing TNF-*α*. Collectively, we firstly proved that LncRNA NONRATT021972 contributed to neuropathic pain of type 2 diabetes in the clinical scenario, while inhibition of LncRNA NONRATT021972 might be a potential therapy to alleviate neuropathic pain.

Previous reports indicated that LncRNA NONRATT021972 was involved in type 2 diabetes and mostly induced hypersensitivity of nervous system, which finally resulted in neuropathic pain [20–22]. Moreover, animal studies have showed that LncRNA NONRATT021972 aggravated diabetic neuropathic pain via P2X3 or other P2 receptors in dorsal root ganglia [23, 24]. However, as described above, such studies were mainly focused on animal studies or underlying mechanisms, while no concrete data were offered in the clinical scenario. Our study showed that increased LncRNA NONRATT021972 was positively associated with aggravated diabetic neuropathic pain, and LncRNA NONRATT021972 might be a potential biomarker for diagnosis or prediction of neuropathic pain among patients with type 2 diabetes. ELISA of the blood samples indicated that LncRNA NONRATT021972 could enhance inflammation via TNF-*α*-related pathways, which was consistent with that of reported animal studies [24, 25]. Neuropathic pain questionnaire (NPQ) was used to collect data of neuropathic pain [26]. In our study, NPQ was modified to adjust enrolled Chinese patients with reported protocols, which had more efficacy of scoring.

We further performed an animal study to testify our result from clinical data and explore whether inhibition of LncRNA NONRATT021972 alleviated neuropathic pain. Consistent with clinical data, STZ-induced diabetic rats indeed had a significantly higher level of LncRNA NONRATT021972 and increased TNF-*α*. What is more, LncRNA NONRATT021972 siRNA decreased mechanical withdrawal threshold and the thermal withdrawal latency of STZ-induced diabetic rats, suggesting that inhibition of LncRNA NONRATT021972 could alleviate neuropathic pain. Our study gave a clue that inhibition of LncRNA NONRATT021972 might be a feasible way to relieve symptoms of neuropathic pain for patients with type 2 diabetes.

## 5. Conclusion

In summary, LncRNA NONRATT021972 was positively associated with neuropathic pain scoring in patients with type 2 diabetes. LncRNA NONRATT021972 exacerbated neuropathic pain via TNF-*α*-related pathways. Inhibition of LncRNA NONRATT021972 could alleviate neuropathic pain.

## Figures and Tables

**Table 1:** Analysis of blood glucose and NONRATT021972 relative expression. D2M group: diabetes patients group. ^∗^*P* < 0.05 versus control group.

Grouping	Blood glucose	NONRATT021972 relative expression
Control group	5.12 ± 1.023	1.00 ± 0.107
D2M group	13.27 ± 2.972^∗^	2.98 ± 0.693^∗^

**Table 2:** Verification of NPQ examination by two coefficients.

	Cronbach's alpha coefficient	Guttman split-half coefficient
NPQ	0.845	0.856

**Table 3:** Analysis of mean NPQ score and NONRATT021972 relative expression in patients without diabetes but have neuropathy.

Grouping	Mean NPQ score	NONRATT021972 relative expression
Control group	2.07 ± 1.02	1.00 ± 0.107
DPN group	9.97 ± 2.38	1.38 ± 0.376
SFN group	8.37 ± 3.43	1.21 ± 0.421
LN group	9.97 ± 3.87	1.17 ± 0.129
